# Molecular mechanisms of pruritus in prurigo nodularis

**DOI:** 10.3389/fimmu.2023.1301817

**Published:** 2023-11-23

**Authors:** Yixin Shao, Duoqin Wang, Yiqi Zhu, Zijing Xiao, Taiyu Jin, Lisi Peng, Yanyun Shen, Hui Tang

**Affiliations:** Department of Dermatology, Huashan Hospital, Fudan University, Shanghai, China

**Keywords:** prurigo nodularis, pruritus, pruritogen, pathogenetic mechanism, therapeutic target

## Abstract

Pruritus is the most common symptom of dermatological disorders, and prurigo nodularis (PN) is notorious for intractable and severe itching. Conventional treatments often yield disappointing outcomes, significantly affecting patients’ quality of life and psychological well-being. The pathogenesis of PN is associated with a self-sustained “itch-scratch” vicious cycle. Recent investigations of PN-related itch have partially revealed the intricate interactions within the cutaneous neuroimmune network; however, the underlying mechanism remains undetermined. Itch mediators play a key role in pruritus amplification in PN and understanding their action mechanism will undoubtedly lead to the development of novel targeted antipruritic agents. In this review, we describe a series of pruritogens and receptors involved in mediating itching in PN, including cytokines, neuropeptides, extracellular matrix proteins, vasculogenic substances, ion channels, and intracellular signaling pathways. Moreover, we provide a prospective outlook on potential therapies based on existing findings.

## 1 Introduction

Prurigo nodularis (PN) is a relatively uncommon dermatosis characterized by recalcitrant chronic pruritus and keratotic nodules (1). It predominantly affects middle-aged and older individuals and Africans (2). Multiple discrete nodules are often symmetrically distributed in scratchable areas, such as the extensor surfaces of the limbs and trunk, with fewer lesions on the harder-to-reach mid-upper back, creating the classical butterfly sign (3). The pruritus associated with PN is notably intense. A previous study has shown that PN exerts a more significant impact on quality of life and carries a higher risk of psychological disorders (e.g., anxiety and depression) than other pruritic dermatoses (4). Notably, approximately half of the patients with PN exhibit coexistent atopic dermatitis (AD) or atopic predisposition (2, 5). This finding implies a potential overlap between the pathogeneses of PN and AD. Although AD and PN are both type 2 inflammatory diseases, recent transcriptomic studies have clearly revealed that PN is separated from AD. PN does not harbor the strong type 2 response pattern that is typically found in AD but is rather characterized by stromal remodeling and neurovascular dysregulation (6–11). Indeed, traditional treatments for AD, including topical steroids and antihistamines, exhibit limited efficacy against PN (1, 12). This underscores the likelihood of PN harboring distinct and yet to be elucidated pathophysiological underpinnings.

However, its exact pathogenesis remains unknown. Current understanding suggests that a persistent “itch-scratch” vicious cycle, leading to recurrent skin excoriation, crusting, and thickening, is the primary driver of nodule formation (3). Skin neuroimmune interactions and neuronal sensitization play important roles in mediating chronic itch in PN (1). Histopathologically, lesional PN skin exhibits epidermal hyperkeratosis accompanied by reduced epidermal nerve fibers (13). Fibrosis, vascular remodeling, and proliferation of afferent nerves are accompanied by mixed inflammatory cell infiltration in the dermis, including T lymphocytes, dendritic cells, mast cells, eosinophils, basophils, and macrophages (14). Notably, mast cells and eosinophils often aggregate around the peripheral sensory nerve endings, establishing close contact with them (15–17).

At the molecular level, the interactions between immune cells and sensory neurons require pruritogens and their receptors to act as intermediaries (18, 19). Pruritogens not only transmit itch by directly stimulating skin sensory neurons but also activate immune cells to release other itch mediators and indirectly stimulate sensory neurons to induce itch (20). Simultaneously, activated sensory neurons secrete pruritogens such as neuropeptides. On one hand, these neuropeptides act on the neurons themselves to increase their own sensitivity and spontaneous activity; on the other hand, they reciprocally activate immune cells and sustain and amplify inflammatory responses, thus promoting chronic itch (21). Ultimately, cutaneous C fibers project itch impulses centrally through pseudo-unipolar dorsal root ganglia (DRG) neurons to the dorsal horn of the spinal cord, which then send projection fibers to the brain (22).

**Figure 1:**
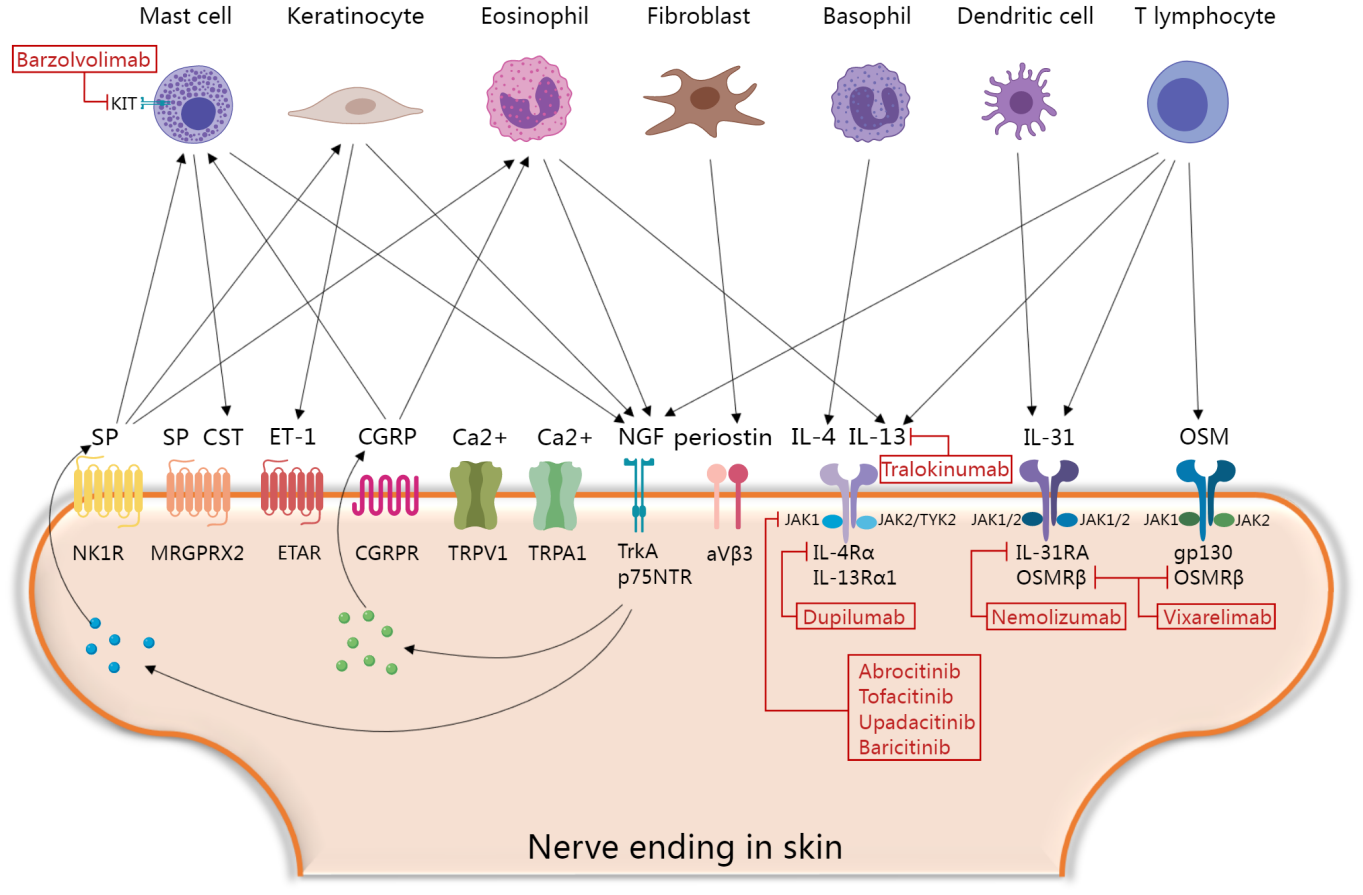
Molecular mechanisms of itch and current and promising therapeutic targets in prurigo nodularis. Skin immune cells, keratinocytes and fibroblasts secrete various histamine-independent itching mediators that directly or indirectly activate corresponding receptors and channels in skin nerve endings, and then promote the secretion of neuropeptides to form positive feedback loops. Finally, the skin-neuroimmune crosstalk mediating the “itch-scratch” vicious cycle of PN. Blocking this process is a key point of novel therapeutic interventions. CGRP, calcitonin gene-related peptide; CST, Cortistatin; ET-1, Endothelin-1; ETAR, the endothelin A receptors; IL, interleukin; JAK, janus kinases-signal transducer; MRGPRX2, Mas-related G protein-coupled receptor member X2; NGF, nerve growth factor; NK1R, neurokinin 1 receptor; OSM, oncostatin M; p75NTR, p75 neurotrophin receptor; SP, Substance P; TrkA, tropomyosin receptor kinase A; TYK2, tyrosine kinase 2; TRP, transient receptor potential; TRPA1, TRP ankyrin 1; TRPV1, TRP vanilloid. The figure was created with MedPeer (medpeer.cn).

Several upregulated pruritogens have been detected within PN lesions (1), including cytokines, neuropeptides, extracellular matrix proteins, and vasculogenic mediators (**Table 1**). Targeting these pruritogens and their receptors is of interest for the development of emerging therapeutics for PN (**Figure 1**). This review aimed to provide a comprehensive summary of the roles and clinical significance of known pruritogens and receptors in the pathogenesis of pruritus in PN.

**Table 1:** Non-histaminergic itch mediators and their cognate receptors and channels in prurigo nodularis.

Pruritogen	Receptor	JAK-STAT pathway		TRP channel	Evidence in PN-related pruritus
IL-4, IL-13	IL-4Rα/y-chain, IL-4Rα/IL-13Rα1, IL-13Rα2	JAK1, JAK3, TYK2, JAK2	STAT3,6	IL-13/TRPA1	Plasma: IL-13↑, skin lesions: IL-4, IL-13 and IL-4R↑, and IL-4 expression is correlated with itching severity.
IL-31	IL-31Rα/OSMRβ	JAK1, JAK2	STAT1,3,5	TRPV1, TRPA1	Serum: IL-31↑, skin lesions: IL-31, IL-31Rα and OSMRβ↑, the intensity of itching is related to the number of IL-31+ cells and IL-31Rα+ cells.
OSM	OSMRβ/gp130, LIFR/gp130	JAK1, JAK2	STAT1,3,5	TRPV1, TRPA1	Skin lesions: OSM↑, the intensity of itching is related to the number of OSM+ cells.
IL-17	IL-17R	/	/	TRPV4	Skin lesions: IL-17 and Th17 cells↑.
IL-22	IL22R1/IL10R2, IL22RA2	JAK1, TYK2	STAT1,3,5	/	Th22/IL-22 polarization in blood and lesional skin.
TSLP	TSLPR/IL-7Rα	JAK1, JAK2	STAT1,3,5	TRPA1, TRPV4	Skin lesions: TSLPR↑.
IL-6	IL-6R/gp130	JAK1, JAK2, TYK2	STAT1,3	TRPV1, TRPA1	Serum: IL-6↑ and is associated with itching severity. skin lesions: gene expression of IL-6↑.
Periostin	Integrin receptor αvβ3	/	/	TRPV1, TRPA1	Plasma: periostin↑, skin lesions: periostin↑ and is associated with the severity of itching.
SP	NK1R, MRGPRX2	/	/	NK1R/TRPV1	Serum: SP↑. skin lesions: the density of SP+ nerve fibers, NK1R and MRGPRX2↑, and MRGPRX2 is correlated with itching severity.
CGRP	CGRP receptor	/	/	/	Dermis: the density of CGRP+ nerve fibers↑.
Cortistatin	MRGPRX2	/	/	/	Skin lesions: cortistatin↑. Ditto for MRGPRX2.
NGF	TrkA, p75NTR	/	/	TrkA/TRPV1	Skin lesions: NGF↑. TrkA and p75NTR↑ in dermal nerve fibers.
VEGF	VEGFR	JAK2	STAT3	TRPV1	VEGF↑ in both skin lesions and serum and correlates with the disease severity.
ET-1	ETAR, ETBR	/	/	TRPA1	ET-1↑ in both skin lesions and serum. ETBR↓ in skin lesions

OSM, oncostatin M; TSLP, thymic stromal lymphopoietin; SP, Substance P; CGRP, calcitonin gene-related peptide; NGF, nerve growth factor; VEGF, vascular endothelial growth factor; ET-1, Endothelin-1; LIFR, leukemia inhibitory factor receptor; NK1R, neurokinin 1 receptor; MRGPRX2, Mas-related G protein-coupled receptor member X2; TrkA, tropomyosin receptor kinase A; p75NTR, p75 neurotrophin receptor; JAK-STAT, janus kinases-signal transducer and activator of transcription proteins;TYK2, tyrosine kinase 2; TRP, transient receptor potential; TRPA1, TRP ankyrin 1;TRPV1, TRP vanilloid 1.

## 2 Pruritogens

### 2.1 Cytokines

#### 2.1.1 Interleukin-4 and IL-13

Although less pronounced than that in AD, T helper (Th)2 immune bias remains a significant characteristic of PN (23). IL-4 and IL-13 are the key cytokines that initiate and sustaining Th2 responses (24). Elevated levels of circulating plasma IL-13 were observed in PN (25). The expression of IL-4, IL-13, and IL-4 receptor (IL-4R) is upregulated in prurigo nodules, with IL-4 correlating with the intensity of itch (8, 11, 26, 27). IL-4 is primarily expressed in T lymphocytes and basophils, whereas IL-13 is primarily expressed in eosinophils (26). Studies have shown that IL-4 and IL-13 activate itch-sensitive neurons in both humans and mice through IL-4Rα (28). They also amplify neuronal responses to various pruritogenic stimuli, including histamine-dependent and histamine-independent pathways, thereby contributing to neuronal sensitization (29). In addition, IL-4 and IL-13 exert pruritogenic effects by recruiting eosinophils and mast cells, driving IgE synthesis, and interacting with IL-31 (30). They promote fibroblast proliferation, differentiation, and synthesis of extracellular matrix proteins such as periostin in the dermis, mediating PN-associated skin fibrosis and itch (20). Although the elicitation of itching by these cytokines has not been reported in humans, they induce pruritus in mice as acute pruritogens (31). Moreover, the combined intradermal injection of IL-4 and IL-13 in mice triggers earlier and more intense scratching behaviors than separate cytokine administration (31). These findings highlight the crucial role of IL-4 and IL-13 in promoting dysregulation of the skin neuroimmune network.

#### 2.1.2 IL-31

IL-31 belongs to the IL-6 cytokine family, which can directly trigger pruritus in humans and is called “itch cytokine” (32). IL-31 is mainly secreted by Th2 cells, and dermal CD11c^+^ myeloid dendritic cells are an important source of IL-31 in PN (33, 34). In contrast to acute pruritogens, itch induced by IL-31 exhibits pronounced delayed characteristics, suggesting its potential for indirect pruritogenic actions, such as regulating the synthesis and release of central itch mediators like BNP in the DRG and skin, and induction of leukotriene B4 production in keratinocytes (35–37). IL-31 is upregulated to varying degrees in various pruritic dermatoses (38). Among them, the upregulation was most remarkable in lesional PN skin, reaching levels 50 times higher than those in healthy skin. Conversely, non-pruritic dermatoses, such as non-pruritic psoriasis, showed no elevation in IL-31 (39). Serum IL-31 levels are also increased in patients with PN (40). The receptor of IL-31 is a heterodimer composed of IL-31 receptor A (IL-31Rα) and oncostatin M receptor β chain (OSMRβ). It is expressed in various cell types including keratinocytes, immune cells, and sensory neurons (32). In PN lesions, this receptor is also notably increased, and the intensity of pruritus in PN is closely related to the number of IL-31+ cells and IL-31Rα+ cells in the dermis (33). IL-31 promotes sprouting of skin sensory nerve axons and enhances their sensitivity to pruritogens (41, 42). Transcriptomic changes in lesional skin and changes in plasma proteomics confirmed the pivotal upstream pathological role of IL-31 signaling in PN (23, 43, 44). Blocking IL-31Rα not only effectively inhibits pruritus signaling but also alleviates downstream Th2 and Th17 inflammatory responses, suppresses nerve growth factor (NGF)-mediated neuronal dysregulation, and reverses the activation of keratinocyte proliferation and pro-fibrotic reactions (23, 43, 44). These findings indicate that the IL-31/IL-31Rα axis plays an important role in the pathogenesis of PN-associated pruritus and involves intricate regulation within the epidermal-immune-neural network.

#### 2.1.3 Oncostatin M

Oncostatin M (OSM) is a member of the IL-6 cytokine family that plays a significant role in various pathological processes in the skin, including inflammation, hyperkeratosis, and fibrosis (45, 46). OSM is upregulated in skin lesions of various pruritic dermatoses including PN (7, 47). Furthermore, the intensity of pruritus in PN is closely correlated with the number of dermal OSM+ cells but not of OSMRβ+ cells (33). Single-cell sequencing has revealed that OSM is primarily produced by dermal T cells and monocytes (42). Similar to IL-31, OSM induces delayed itching in mice and enhances histamine-induced pruritus by enhancing the excitability and sensitivity of the sensory neurons to pruritogens instead of directly activating them. OSM also indirectly induces itch by stimulating stromal cells in the skin (42). OSM has two types of receptors, the unique human type I receptors consisting of leukemia inhibitory factor receptor (LIFR) and gp130, and the type II receptors found both in humans and mice composed of gp130 and OSMRβ (45). It has been reported that OSMR is expressed by itch-selective neurotensin B (Nppb) neurons in mice. Knocking out OSMR in sensory neurons or systemic administration of gp130 inhibitors suppresses inflammatory itch in mice (42). This suggests that targeting the OSM/OSMRβ/gp130 signaling pathway holds promising potential for antipruritic therapy. Since OSMRβ is a shared receptor subunit for both OSM and IL-31, antagonizing it might be an effective approach for treating PN.

#### 2.1.4 IL-17

The Th17 cell cytokine IL-17 mediates excessive proliferation and differentiation interference of epidermal keratinocytes and plays a key pathogenic role in conditions such as psoriasis (48). Interestingly, the molecular biological features of PN are more akin to those of psoriasis than those of AD (10). Previous studies have shown an increased expression of IL-17 in lesional PN skin, coupled with an increase in the number of Th17 cells within the lesions (9, 27, 49). The origin of CD4+ Th17 cells is significantly more from PN lesions compared to from healthy skin and lesional AD skin (9). However, the relationship between IL-17 and pruritus in patients with PN remains unclear. IL-17A stimulates keratinocytes to secrete pruritogenic endothelin-1 (49). The latter is upregulated in prurigo nodules (49, 50), implying that IL-17 indirectly contributes to development of itching symptom in patients with PN.

#### 2.1.5 IL-22

Several studies have shown that the expression of the Th22 cell cytokine IL-22 is elevated in pruritic PN lesions, which is potentially associated with impaired epidermal proliferation and differentiation and skin inflammation (27, 51). Skin RNA sequencing of patients with severe pruritus in PN revealed robust upregulation of Th22-related genes and signaling pathways, including IL-22, IL-22 receptors (IL22RA1 and IL22RA2), and IL-22-associated cytokines. Moreover, circulating IL-22 derived from both CD4+ and CD8+ T cells is significantly increased in PN, suggesting a systemic and cutaneous Th22/IL-22 polarization pattern (51). However, there is limited evidence regarding the correlation between IL-22 levels and pruritus in PN. Considering that IL-22 receptors are exclusively expressed in epithelial cells (52), IL-22 may indirectly mediate pruritus by promoting pruritogen secretion. For example, IL-22 induces keratinocytes to express substantial amounts of pruritogenic gastrin-releasing peptides (GRP) and GRP receptors (53).

#### 2.1.6 Other cytokines

Thymic stromal lymphopoietin (TSLP) is primarily produced by epithelial cells and signals through IL-7 receptor α-chain (IL-7Rα) and TSLP receptor (TSLPR) heterodimers (54). TSLP released from keratinocytes activates TSLPR-expressing sensory neurons to trigger itch sensation in mice (55). Although levels of TSLP were not elevated in PN lesions, TSLPRs were upregulated (56). IL-6 is expressed in the dermal nerve fibers of lesional PN skin (57). Transcription level of IL-6 is increased in prurigo lesions (8). Serum IL-6 levels are significantly elevated in patients with PN and are correlated with the severity of pruritus (58). However, direct evidence linking IL-6 and TSLP to pruritus in PN is lacking.

### 2.2 Extracellular matrix protein

#### 2.2.1 Periostin

Periostin is an extracellular matrix protein that plays a significant role in skeletal development, cardiovascular remodeling, and Th2 inflammation (59). Owing to its ability to induce rapid and intense scratching behavior in mice, dogs, and monkeys, it has been identified as a novel pruritogen (60). Enhanced dermal periostin expression has been observed in various pruritic skin disorders (61–63). Similarly, periostin is abundantly deposited in the dermis of PN and is significantly correlated with the severity of pruritus in PN (64). Plasma periostin was also significantly upregulated in patients with PN with severe itch (25). Single-cell sequencing has revealed that the activation of fibrotic responses is a distinguishing feature between PN and AD. The main contributor to dermal fibrosis, the COL11A1+ fibroblast subset, is likely to be the primary cellular source of periostin in PN (23). Periostin-mediated itching involves direct and indirect pathways. In the direct pathway, periostin interacts directly with itch-sensitive nerve fibers via its receptor integrin aVβ3 (60). In the indirect pathway, periostin stimulates immune cells (macrophages, eosinophils, and basophils) to release IL-31 and other pruritogenic mediators (59, 62). Periostin also stimulates keratinocytes to secrete the pruritogen TSLP, which in turn acts on fibroblasts to generate periostin, forming a “TSLP- periostin” cross-activation loop that sustains chronic itching and maintains Th2 inflammation (60). Since dermal periostin in PN shows no significant correlation with other pruritogenic mediators in the dermis (including IL-31, IL-31Rα, and OSMRβ), it might be an independent contributor to pruritus in PN (64). Further studies are required to elucidate this association. Targeting periostin or its receptor integrin aVβ3 to simultaneously treat itch and Th2 inflammation could be a novel therapeutic option for PN.

### 2.3 Neuropeptides

#### 2.3.1 Substance P

Substance P (SP), secreted by sensory neurons, plays an important role in itch signaling within the peripheral and central nervous systems (65, 66). The subcutaneous injection of SP immediately induces itching in both humans and mice (67, 68). The density of SP-expressing nerve fibers was increased in the dermis of prurigo nodules but not in that of neurodermatitis, another pruritic neurogenic skin disorder (69–71). Serum SP levels and the expression of two SP receptors, neurokinin 1 receptor (NK1R) and Mas-related G protein-coupled receptor member X2 (MRGPRX2), are elevated in patients with chronic prurigo (72, 73). Additionally, the expression of MRGPRX2 closely correlates with the severity of itch (73). This evidence suggests a unique role of SP signaling in PN. At the molecular level, SP mediates mast cell activation, leading to the release of pruritogens such as histamine, leukotriene B4, and vascular endothelial growth factor (VEGF) (74–76). However, the pruritogenic effect of mast cells may not be crucial because mast cell-deficient mice still exhibit intense scratching behavior after SP stimulation (67). Keratinocytes and eosinophils activated by SP producing NGF promotes neuronal proliferation and activates afferent nerves, leading to further release of SP and sustaining the itch cycle (77). Moreover, SP directly activates sensory neurons to trigger itch (78). NK1R antagonists have shown preliminary results for the treatment of chronic pruritus in patients with PN. An 8-week phase 2 clinical trial of the NK1R antagonist serlopitant demonstrated a greater reduction in itch with oral serlopitant than with placebo (79). However, phase 3 randomized clinical trial (RCT) of the drug did not meet the primary endpoint of reducing itch (NCT03546816 and NCT03677401). Another NK1R antagonist, aprepitant, in both topical and oral formulations, did not show satisfactory antipruritic effects in placebo-controlled trials involving patients with PN (72, 80). MRGPRX2 antagonization is another potential therapeutic option for PN-associated itching. Therefore, it is necessary to investigate whether NK1R antagonists interact with MRGPRX2. Although aprepitant was incapable of antagonizing human MRGPRX2 *in vitro*, it was found to have an off-target effect on MrgprB2 (a homologous receptor of MRGPRX2) in animal experiments (78, 81).

#### 2.3.2 Calcitonin gene-related peptide

Calcitonin gene-related peptide (CGRP) is the most abundant neuropeptide in human skin and is often colocalized with SP (82). Its function is similar to that of SP (83). However, intradermal injection of CGRP induces persistent erythema, but does not cause itch (84). Evidence suggests that sensory neurons expressing CGRP are necessary for both histamine-dependent and histamine-independent itch (85). CGRP antagonist blocks trypsin-induced itch in mice (86). In lesional PN skin, the density of CGRP-expressing afferent nerves in the dermis was increased, surrounded by mast cells and eosinophils (16). CGRP activates mast cells to release histamine, and eosinophils to produce NGF, another pruritogen. Histamine and NGF, in turn, promote release of CGRP by neurons, creating a bidirectional positive feedback loop between nerve fibers and immune cells; thus, amplifying and sustaining itch signaling (77). In mice, CGRP induces Th2 immune responses, promoting the production of IL-4, CCL17, and CCL22 by Langerhans cells, while suppressing Th1 responses (87). CGRP also acts as an immunoregulatory mediator that enhances IL-13 production (88). Additionally, CGRP influences endorphin levels and leads to the dysregulation of mu- and kappa-opioid receptor expression (3, 89, 90). These factors may contribute to PN-related itching; however, direct evidence of the relevance of CGRP is still lacking.

#### 2.3.3 Cortistatin

Cortistatin (CST) is a neuropeptide that is structurally and functionally similar to somatostatin (91). It possesses various biological effects, such as regulating homeostasis in the nervous, endocrine, and cardiovascular systems, exerting anti-inflammatory effects, and promoting Th2 polarization within the immune system (92). Recently, it was identified as an endogenous pruritogen that is predominantly secreted by skin mast cells. Pricking CST on the skin of healthy individuals rapidly induces a noticeable itch sensation (73). CST and its receptor, MRGPRX2, play a pivotal role in the development of chronic itch (93). CST- and MRGPRX2-expressing cells, mostly mast cells, increased in the skin lesions of patients with chronic prurigo. Severe itch in patients with chronic prurigo is associated with the most significant upregulation of CST in the skin. Moreover, the number of cells expressing MRGPRX2 in skin lesions and serum levels of MRGPRX2 correlate with prurigo severity, itch intensity, and/or impaired quality of life (73). The pruritogenic mechanism of CST may involve binding to MRGPRX2, inducing mast cell degranulation and the release of histamine and CST, thus forming a CST autocrine feedback loop (73). MRGPRX2 is expressed in DRG sensory neurons (93, 94); however, whether CST directly activates neurons through its receptor to induce itching remains to be further elucidated.

### 2.4 Neurotrophin

#### 2.4.1 Nerve growth factor

While keratinocytes are the primary source of skin-derived NGF, dermal inflammatory cells such as mast cells, eosinophils, and lymphocytes abundantly secrete NGF in lesional PN skin (95, 96). The two NGF receptors, high-affinity tropomyosin receptor kinase A (TrkA) and low-affinity p75 neurotrophin receptor (p75NTR), have a synergistic effect and are increased in the dermal nerve fibers of PN (95). Scratching can also lead to increased expression of skin-derived NGF and its receptors (97). NGF is a neurotrophic factor that primarily activates, sensitizes, and sprouts skin nerve fibers, promoting the release of neuropeptides, such as SP and CGRP (96, 98). NGF also influences the survival and function of non-neuronal cells. For example, it stimulates the proliferation and differentiation of keratinocytes; activates or enhances mast cells, eosinophils, and basophils; releases various pro-inflammatory and pruritogenic mediators; and supports the survival and differentiation of these immune cells (96). Intradermal injection of NGF in healthy individuals enhances non-histaminergic itch induced by cowhage (99). Therefore, antagonizing NGF and its receptors may be beneficial for PN treatment; however, this has not been studied in PN.

### 2.5 Vasculogenic substances

#### 2.5.1 Vascular endothelial growth factor

VEGF is produced by various resident skin cells, including keratinocytes (100). Mechanical manipulation may stimulate keratinocytes to produce VEGF. It promotes endothelial cell proliferation and angiogenesis, which may be associated with the formation of prurigo nodules (101). VEGF levels in both skin lesions and serum are increased in patients with prurigo and are correlated with the severity of the condition (10, 100, 101). However, psoriasis or AD lesions do not show increased VEGF activity, suggesting a unique role of VEGF in the pathogenesis of PN (10). A case of simplex prurigo treated with bevacizumab, a monoclonal anti-VEGF antibody, showed significant improvement in itch (101). Further studies are required to determine the role of VEGF in PN-related itching.

#### 2.5.2 Endothelin

Endothelin-1 (ET-1), initially described as a vasoconstrictor, is also a partial non-histaminergic pruritogen that independently induces a persistent itching sensation in human skin (50). In patients with PN, both skin lesions (especially in the epidermis and neurons) and serum levels of ET-1 are increased, which is possibly associated with elevated levels of IL-17 (49, 50). IL-17A induces ET-1 expression in keratinocytes via the p38 MAPK pathway (49). ET-1 signals through two receptors: the endothelin A receptors (ETAR) and endothelin B receptors (ETBR) (21). ET-1 mediates itch through ETAR in the skin nerve fibers; however, this process is negatively regulated by neutral endopeptidase 1 (ECE-1) (50). In contrast, ETBR has an antipruritic effect through peripheral κ-opioid receptors against ET-1-induced itch (102). ETBR expression is downregulated in lesional PN skin (56), further enhancing the pruritic effects of ET-1 signaling.

### 2.6 Histamine

Histamine is one of the most classical pruritogens. Its primary sources are activated mast cells and basophils (103). Immunohistochemistry showed that the number of histamine-containing mast cells in PN lesions was significantly increased, and the morphology of mast cells also changed, with enlarged cell bodies and more dendrites, but fewer intracellular granules (17, 104). Similarly, the number of activated basophils in the blood and dermis of patients with PN was also significantly higher than that in healthy controls (105). Histamine activates cutaneous sensory nerve fibers through H1 and H4 histamine receptors to elicit itch (106). However, due to the resistance of PN to antihistamine treatment, histamine is not considered a primary pruritogenic factor in PN (12).

## 3 Itch signaling pathways

### 3.1 The JAK-STAT pathway

Four members of the Janus kinase (JAK) family (JAK1, JAK2, JAK3, and TYK2) selectively bind in various combinations to different type I/II cytokine receptors and transmit their activated intracellular transcriptional signals, along with seven members of the signal transducer and activator of transcription protein (STAT) family (107). Several key cytokines, such as IL-4, IL-13, IL-31, and OSM, play roles in propagating pruritus and inflammation in PN through the JAK-STAT signaling pathway, particularly involving JAK1, STAT3, and STAT6 (28, 45, 108). Th2 cytokines are predominantly regulated by STAT6, whereas STAT3 is associated with multiple pruritogens, including IL-6, IL-22, IL-31, OSM, TSLP, and VEGF (109–111). STAT3 and STAT6 are significantly upregulated in lesional PN skin (112–114); thus, JAK inhibitors may effectively slow disease progression. Numerous case reports have shown the successful treatment of refractory PN with tofacitinib (JAK1/3 inhibitor), baricitinib (JAK1/2 inhibitor), and upadacitinib (JAK1 inhibitor) (115–124). Phase 2 clinical trials of two JAK1 inhibitors, abrocitinib (NCT05038982) and povorcitinib (NCT05061693), for the treatment of PN, as well as a phase 3 clinical trial of ruxolitinib cream (a JAK1/JAK2 inhibitor) are currently underway (NCT05755438 and NCT05764161).

### 3.2 Transient receptor potential channels

Transient receptor potential (TRP) channels can be activated by various physical and biochemical stimuli. Notably, most itch receptors such as G-protein-coupled receptors and cytokine receptors couple with TRP channels that act as downstream sensors (21). After activation, the channels develop to cause calcium influx and generate action potentials that propagate itch signals in the peripheral sensory neurons (19, 125). TRP channels are a class of nonselective cation channels including family members like TRP vanilloid 1 (TRPV1) and TRP ankyrin 1 (TRPA1) (126). TRPV1, also known as the capsaicin receptor, is significantly upregulated in nerve fibers and keratinocytes within lesional PN skin (127). Propagation of various histamine-independent itch signals related to the PN involves TRPV1 and/or TRPA1. For example, itching induced by IL-31 and periostin is transmitted by TRPV1+TRPA1+ neurons (60, 128). Itch, mediated by IL-13, TSLP, endothelin, and MRGPRs, is activated by TRPA1 (55, 129–131). NGF activates TRPV1 via TrkA, causing the upregulation and activation of TRPV1 in afferent nerves, subsequently releasing SP and CGRP (132). Topical capsaicin treatment alleviates itching symptoms in patients with PN, normalizes TRPV1 expression in skin lesions, and reduces SP and CGRP levels (127). The anti-itch effect of capsaicin is presumably mediated by the activation of TRPV1 on cutaneous C-fibers, leading to the depletion of neuropeptides such as SP (133). However, because of the short-acting efficacy of capsaicin and its side effects of intense burning sensations, its widespread use is limited. Further evidence is required to confirm the roles of TRPV1 and TRPA1 in PN-associated itch.

## 4 Promising advances in the treatment of PN

Based on two phase 3 clinical trials, dupilumab (an IL-4Rα monoclonal antibody simultaneously antagonizing IL-4 and IL-13) has become the first the U.S. Food and Drug Administration (FDA)/European Medicines Agency (EMA)/China National Medical Products Administration (NMPA)-approved treatment for adult patients with PN. Compared to placebo, significant improvements in weekly average worst itch numeric rating scale (WI-NRS) with dupilumab were observed as early as week 3 in PRIME and week 4 in PRIME2 (134). Moreover, a few cases report off-label use of dupilumab have shown good efficacy and safety in children and adolescents with PN (135–137). It took longer for dupilumab to reduce pruritus in atopic PN compared with patients with nonatopic PN (138). Two real-world studies reviewed the long-term efficacy of dupilumab. Among 19 patients with PN, 78.9% and 68.4% reported improved pruritus at weeks 16 and 52, respectively (139). The pruritus NRS score dropped to 0 at 16 weeks of treatment in 19 of 21 PN patients and was maintained for at least 104 weeks (140). A systematic review showed that 48.88% of patients with PN achieved complete relief of itching, the average time to clear itching was 19 weeks, and patients who did not achieve complete itch resolution had a longer time to first relief (138). Large real-world data collections evaluating the efficacy and safety of dupilumab in PN are still lacking.

In addition to dupilumab, a series of novel drugs targeting Th2 polarization in PN have also shown promising results. In an open-label case series, 17 patients with a PN-like phenotype of AD showed a significant reduction in mean itch-NRS values as early as week 4 after treatment with the anti-IL-13 monoclonal antibody tralokinumab (141). Recently, phase 2 and phase 3 clinical trials on nemolizumab, an anti-IL-31Rα monoclonal antibody, demonstrated the effectiveness and safety of blocking the IL-31 signaling in treating PN. Both studies reported a rapid reduction in pruritus severity and significant improvement in nodules after treatment (142, 143). The OSMRβ monoclonal antibody, vixarelimab (KPL-716), has shown promising results in a phase 2a clinical trial for PN (45). They demonstrated an average reduction of 50% in pruritus by week 8 of vixarelimab treatment, and one-third of the patients achieved lesion clearance or near clearance (45). A phase 2b trial is currently in progress (NCT03816891).

IL-5 plays a central role in the differentiation, proliferation, activation, adhesion, and survival of eosinophils, while promoting the recruitment of skin mast cells and basophils (19, 144). A phase 2 clinical trial of anti-IL5Rα antibody (benralizumab) for PN treatment is currently in preparation (NCT05528913). Blocking mast cell activation suppresses the neuro-immune axis (145, 146). Targeting mast cell tyrosine kinase KIT receptors (barzolvolimab) results in sustained and profound MC inhibition in healthy volunteers (147). A phase 1 clinical trial targeting PN with this drug has been completed and the results are awaited (NCT04944862).

Several recent omics studies have suggested that extracellular matrix remodeling, fibrosis activation, neural dysfunction, vascular system development, and keratinization may be unique pathological features of PN (6–8, 23, 43, 148). Further investigation of the relationship between these features and itching is required. Therefore, extracellular matrix proteins, such as periostin; neuroinflammatory molecules, such as neuropeptides, NGF, and MRGPRX2; and vascular substances, such as VEGF and ET, are intriguing targets for drug development. Likewise, blocking downstream cellular signaling pathways, such as with JAK inhibitors and TRP channel antagonists, could be an additional treatment option. Several JAK inhibitors have been approved for moderate-to-severe AD in many countries, and they have significantly improved patients’ pruritus and condition (149). Topical TRPV1 antagonists have demonstrated efficacy and safety in phase 2b (PAC-14028) and phase 3 (Asivatrep) clinical trials for treating atopic itch (150, 151). However, their role in the treatment of PN requires confirmation through RCT.

Cannabinoid receptors 1 and 2 (CB1 and CB2) are expressed in the central nervous system and skin nerve fibers, and are activated by various bioactive lipid mediators (152). Cannabinoid agonists alleviate pruritus in various animal models of chronic pruritus and in clinical trials of pruritic skin diseases (153–158). An open-label clinical study showed significant relief from itch in eight of 12 patients with PN using a moisturizing cream containing the CB2 agonist N-palmitoyl ethanolamide (159). However, the role of the endogenous cannabinoid system in PN requires confirmation through double-blind controlled trials. At the level of the spinal cord, an imbalance between itch-promoting μ-opioid receptor (MOR) activity and itch-inhibiting κ-opioid receptor (KOR) activity mediates non-histaminergic itch (160). A phase 2 placebo-controlled study indicated that the dual-acting KOR agonist/MOR antagonist nalbuphine extended-release (ER) tablets effectively treat PN. Furthermore, 33% of participants treated with 162 mg oral doses twice daily showed a reduction of ≥50% in itch by week 10, with a suitable safety profile (161). The phase 2b/3 PRISM clinical trial of nalbuphine ER reached its primary endpoint, with a greater proportion of participants achieving a ≥4-point reduction in WI-NRS at week 14 compared to placebo (24.7% *vs* 13.9%) (156). The new and emerging treatments for PN-related pruritus are summarized in **Table 2**.

**Table 2:** Emerging therapies for the treatment of prurigo nodularis.

Drug	Target	Status	Key clinical data	References
Systemic treatment				
Dupilumab	anti-IL-4Rα	Approved for adult patients with PN	A ≥4-point worst-itch NRS reduction at week 24 in the dupilumab and placebo arms was achieved by 60.0% and 18.4% of patients, in PRIME (P < 0.001) and at week 12 by 37.2% and 22.0% of patients, respectively, in PRIME2 (P = 0.022).	Yosipovitch G, et al., 2023 (134)
Nemolizumab	anti-IL-31α	Phase 3	At week 16, 56% of nemolizumab-treated patients achieved an ≥4-point reduction in itch, as measured by the PP-NRS score, compared to 21% of placebo group (P < 0.0001).	NCT05052983 NCT04501666 NCT04204616 NCT04501679 Galderma, 2022 (45)
		Phase 2	At week 4, the PP-NRS was reduced from baseline by 4.5 points versus 1.7 points in the nemolizumab and placebo group (P < 0.001).	Ständer S, et al.2020 (142)
Vixarelimab	anti-OSMRβ	Phase 2a	At week 8, LS-PCFB in worst-itch NRS score was -50.6% versus -29.4% in the vixarelimab and placebo group (P = 0.03)	Sofen H, et al.2023 (162)
Tralokinumab	anti-IL-13	Open application observation	17 patients with PN-like phenotype AD show a significant reduction in mean itch-NRS values as early as week 4	Pezzolo E, et al.2023 (141)
Benralizumab	anti-IL5Rα	Phase 2	No data available yet	NCT05528913
Barzolvolimab	Tyrosine kinase KIT receptor	Phase 1	No data available yet	NCT04944862
Abrocitinib	JAK1 inhibitor	Open application observation	Significantly reduced 78.26% of PP-NRS from baseline to week 12 (P < 0.001)	NCT05038982
Tofacitinib	JAK1 and JAK3 inhibitor	Case series	Nine patients experienced significant relief of pruritus 1 week	Liu T, et al.2023 (117)
Upadacitinib	JAK1 inhibitor	Case series	Three patients reported rapid itch improvement within 3-7 days	Gil-Lianes J, et al.2023 (119)
Baricitinib	JAK1 and JAK2 inhibitor	Case reports	Three patients reported significant itch relief within 1-12 weeks	Yin M, et al.2022 (120) Pereira MP, et al.2022 (121) He Y, et al.2021 (122)
Povorcitinib	JAK1 inhibitor	Phase 2	No data available yet	NCT05061693
Serlopitant	NK1R antagonist	Phase 3	Non-significant difference in worst-itch NRS at week 10 in both serlopitant and placebo group in two trials	NCT03677401 NCT03546816
		Phase 2	Significantly greater decrease from baseline in average itch NRS scores in the serlopitant group at week 2 versus placebo group (P = 0.009)	NCT03540160
Nalbuphine	KOR agonist and MOR antagonist	Phase 2b/3	Significantly more participants responding to nalbuphine with a ≥4-point reduction in the worst-itch NRS compared with placebo at week 14 (24.7% *vs* 13.9%; P = 0.0157).	NCT03497975 Ständer S, et al., 2022 (163)
		Phase 2	Itch reduction was significant among 66.7% subjects completing week 10 treated with nalbuphine *vs*. placebo (40.0%; P = 0.03).	NCT02174419
Topical treatment				
PEA	Cannabinoid receptor 2 agonist	Open application observation	Significant relief in itching for 8/12 patients using a moisturizing cream containing PEA.	Ständer S, et al. 2006 (159)
Aprepitant	NK1R antagonist	Phase 2	No significant differences were found between aprepitant treatment and placebo for any of the parameters investigated.	Tsianakas A, et al. 2019 (80)
Ruxolitinib	JAK1 and JAK2 inhibitor	Phase 3	No data available yet	NCT05755438 NCT05764161

PP-NRS, peak-pruritus numerical rating scale; LS-PCFB, least squares-mean percent change from baseline; OSMRβ, oncostatin M receptor β chain; NK1R, neurokinin 1 receptor; KOR, κ-opioid receptor; MOR, μ-opioid receptor; PEA, N-palmitoyl ethanolamide.

## 5 Conclusion

Recent studies have provided strong evidence for unraveling the specific mechanisms underlying itching in PN. Further exploration of independent pruritogens and receptors, as well as investigation of the interactive network between skin cells and the nervous system, is crucial for a comprehensive understanding of PN. Targeting pruritus signal transmission or disrupting neuroimmune crosstalk are emerging therapeutic strategies to alleviate itching, prevent chronicity, and improve disease prognosis in PN.

## Author contributions

YXS: Conceptualization, Investigation, Methodology, Visualization, Writing – original draft, Writing – review & editing. DW: Writing – original draft, Writing – review & editing. YZ: Writing – review & editing. ZX: Writing – review & editing. TJ: Writing – review & editing. LP: Writing – review & editing. YYS: Writing – review & editing. HT: Conceptualization, Funding acquisition, Resources, Supervision, Writing – review & editing.
